# Delamination Strength Comparison of Additively Manufactured Composite Curved Beams Using Continuous Fibers

**DOI:** 10.3390/polym15193928

**Published:** 2023-09-28

**Authors:** Sedat Süsler, Zafer Kazancı

**Affiliations:** 1 Faculty of Aeronautics and Astronautics, Kocaeli University, Kartepe 41285, Kocaeli, Turkey; s.susler@qub.ac.uk; 2 Advanced Composites Research Group, School of Mechanical and Aerospace Engineering, Queen’s University Belfast, Belfast BT9 5AH, UK

**Keywords:** curved beam, delamination, four-point bending, continuous fiber, additive manufacturing, continuous filament fabrication

## Abstract

The objective of this study is to show the applicability of various 3D-printed composite curved beams using continuous fibers and their delamination strength when they are subjected to bending loading. Four-point bending tests are configured for comparative research on evaluating the effect of fiber types on the delamination strength and failure mode. Out-of-plane tensile properties are calculated analytically by using experimental data. The number of curved beams per build during multiple printing is examined to observe the effect of delay time between each deposited layer of parts. Macro-scale finite element simulations including surface-based cohesive concept for the selected 3D-printed composite curved beam design are also presented and compared. The analytical results show that carbon fiber reinforced curved beam design is superior to the other fiber types by at least 18% in the interlaminar tensile strength and is relatively challenging against the conventionally manufactured composite curved beams in the literature despite its low fiber volume ratio. There is no gross effect of delay time between each deposited layer of parts, although printing a single sample is favorable for better strength. There is a presence of compatibility between the analytical and numerical results as the percentage difference for maximum load, radial tensile strength and maximum displacement are found as 1.8%, 2.4% and 1.5%, respectively, in a 3D cohesive model. A 2D cohesive model offers a fast solution and a competitive agreement with test results when the 2D and 3D finite element models are compared.

## 1. Introduction

Composite materials are growing in demand due to their superior capabilities to meet the requirements for lightweight advanced structures in the aerospace industry. Although the manufacturing of particle or discontinuous fiber-reinforced composites can be faster and easier, the continuous fiber filament and the woven fabric stand out from the rest in their capability. Conventional composite manufacturing methods, which are based on using molding, vary from simple to advanced and from a low to high fiber volume ratio (*V_f_*). The shape of the structure and the complexity of geometry also designate the production method. A *V_f_* can be achieved of up to 0.70 when pre-impregnated fabrics (prepreg) and autoclaves are used [1]. The main disadvantages, which can cover all the conventional methods, are the close relationship between the product quality and the experience of a manufacturer, the cost of molds and tools especially for complex structures to obtain high *V_f_*, limitations for complex geometries and long production time. These disadvantages could even result in an untenable situation, if rapid prototyping was needed after a comprehensive engineering analysis. It seems that additive manufacturing (AM), which is one of the most popular and fastest developing technology topics today, could be a shoo-in to overcome these disadvantages of manufacturing composite structures in the future. The AM industry, which had a market size of USD 13 billion for equipment and service only in 2020, will quadruple in 2024 [2]. While AM is gradually advancing towards the goal of the print-it-all concept [3] of advanced large structures, it is also on the threshold of a new era after the beginning of using continuous fibers in the fused deposition modelling (FDM) based AM. This recent technique, referred to fused filament fabrication (FFF), has enabled the fabrication of fiber-reinforced thermoplastic matrix 3D-printed composites with a high resolution to create complex and custom-designed structures that would be inconvenient or unfeasible to manufacture using conventional composite fabrication methods. In recent years, using AM techniques to attempt to produce composite structural aerospace components such as brackets has been consequently attractive.

A bracket, which is generally in the form of an L-shaped structural part, involves at least a critically loaded 90° bend. Brackets have a wide range of areas of utilization, especially in the aerospace industry to support and reinforce the various systems and components of an aircraft, such as its fuselage, wings and engines. The longitudinal and transverse structures of semi-monocoque aerospace fuselages and wings as well as wind turbine blades are not monolithic and are mainly assembled via brackets. In addition to the act of a fitting, there are crack-stoppers and stiffeners in the form of brackets for some structures of large aircraft subjected to high stresses. Although titanium and aluminum alloys (AAs) such as AA7075 or AA6061 have commonly become primary materials for manufacturing brackets, researching the availability of brackets made of advanced composites is the current trend and this will bring forth replacing metallic brackets with composite ones.

The curved region of a laminated composite bracket obviously attracts considerable attention in the literature as it is prone to delamination when the assembled structure such as a wing spar is subjected to its primary loading conditions that cause the bracket to be loaded with flexural moments. Therefore, delamination, which is the most destructive damage mode for composites, should be analyzed to withstand the demanding conditions of the aerospace environment. The delamination strength of curved composite laminates is the primary concern in early-stage research of bracket design and production. An L-shaped curved beam with a single curved region can represent a sophisticated aerospace bracket as a simplified model that captures its essential features and behaviors while abstracting away from the less important or irrelevant details. The methodology of ASTM D6415/D6415M-22 standard, which depends on a four-point bending test of a fiber-reinforced plastic curved beam, offers less complexity as a mechanical test procedure and the preparation of a test coupon to measure the delamination strength. In addition, the method is effective for obtaining the curved beam strength (CBS) and interlaminar tensile failure data directly for structural design and analysis. The test setup minimizes interlaminar shear stress via its capability of producing interlaminar tensile stress by creating a bending moment in the bend [4].

In the literature, there have been many experimental and numerical studies, which are related to the four-point bending test of a composite curved beam, especially over the last 10 years. Zou et al. [5] investigated various laminated composite curved beams with different orientations experimentally, as well as numerically by using cohesive elements, cohesive surfaces and perfect bonded interfaces. Wu et al. [6] examined a composite curved beam design that contained an internal de-icing system and showed the effect of the internal heat on the delamination occurring in the curved region. Qian et al. [7] discussed the effects of out-of-plane material properties on the failure mode of composite L-shaped structures significantly and investigated the effect of gap and overlap on the out-of-plane tensile strength of the structure. Luinge and Warnet [8] investigated the applicability of replacing recycled chopped thermoplastic laminates after stamp forming as a middle layer of a laminated beam, which is subjected to bending and bearing load, as well as a curved beam test for the use of an aerospace bracket. Ranz et al. [9] proposed an improved cohesive zone numerical model that took account of the fiber bridging and the change in the element size in the bend for the better agreement of delamination in carbon/epoxy curved beams with experimental results. Xu et al. [10] investigated the effect of out-of-plane wrinkles as a defect due to manufacturing curved carbon/epoxy structures on the interlaminar tensile strength and found an up to 16% reduction in strength. The effects of other defects such as fiber misalignment, cracks and voids on progressive delamination behavior under the four-point bending test for carbon/epoxy laminated L-shaped beam were investigated by Woo et al. [11]. Petersen et al. [12] examined multi-directional laminated composite curved structures by an improved Lekhnitski formula [13] and validated their results with high resolution strain measurements as the Lekhnitski formula is valid for unidirectional (UD) laminated materials only. Ranz et al. [14] conducted experimental studies on carbon/epoxy laminated curved beams to focus on the benefits of using acoustic emission techniques in detecting delamination in the curved region. There are also studies that concentrated on increasing the loading capacity or CBS via distinctive applications to the curved region. Arki et al. [15] introduced a modified shape, which contained a flat region, for the bend of a laminated composite curved beam and achieved a 15% increase in strength by comparing it with the conventional curved region of the standard. Moreover, Ranz et al. [16] in their research on carbon/epoxy laminated curved beams with various thicknesses, identified the region where delamination occurred and then reinforced that region via stitching a fiber through the thickness with different densities. Ju et al. [17] performed the reinforcement process through the thickness with grooved steel Z-pins in the curved region and obtained up to a 42% increase in strength depending on the diameter of the pins and the number of pins in a unit area. On the other hand, Fletcher et al. [18] increased the strength of a curved laminated beam by 16% as a result of reducing the edge effect and delaying the delamination via applying resin to the free edges. Arca et al. [19] focused on reinforcing the epoxy resin with various carbon nanotube fractions to increase the strength of carbon/epoxy curved beams that were manufactured by a hand layup technique. Another distinctive investigation is the effect of pre-impact damage applied to the carbon/epoxy curved region on CBS and maximum interlaminar tensile stress by Hao et al. [20]. 

Studies in the literature above concerned the degradation and strength of conventionally molding-based manufactured composite curved beams under four-point bending. In recent years, the fiber-reinforced composite AM technology has been developing, and it has been making great progress on various mechanical behaviors of additively manufactured composites. Some of the studies about 3D-printed continuous fiber-reinforced composites and their mechanical properties were critically reviewed by Kabir et al. [21] and Handwerker et al. [3]. In further studies about compressive properties [22], Mode I and Mode II interlaminar fracture toughness [23,24,25,26,27] and interlaminar shear strength obtained by the short beam test [27,28,29,30,31] were investigated. Fiber-reinforced 3D-printed composites in those studies are in the form of flat beams and do not indicate analyzing out-of-plane tensile stress. To the author’s knowledge, the applicability of composite AM and the potential effects of continuous filament fabrication (CFF), which is an enhanced FFF process that additionally works for laying continuous fibers, have not been studied yet on the delamination strength of composite curved beams by the four-point bending test as well as by any other curved beam test methods. The curved beams in the previous studies might not represent the behavior of a 3D-printed curved beam as fiber-reinforced AM, which is a new phenomenon for composite production methods and has a completely different manufacturing mentality that leads to different material properties and structural failure characteristics. The results in analyzing the delamination strength of 3D-printed composite curved beams made of various fiber materials would offer helpful information for further research to enhance composite brackets.

In this comparative study, additively manufactured continuous fiber-reinforced thermoplastic curved beams subjected to bending loading are investigated experimentally and numerically. Four-point bending tests are carried out to evaluate the effect of fiber types on the strength and failure mode of composite curved beams. Load-displacement, curved beam moment per unit width-displacement and maximum interlaminar tensile stress (MILTS)-displacement histories are obtained for all specimens. Maximum load, CBS and interlaminar tensile strength (ILTS) at a certain radial location in the curved region of each type of fiber-reinforced beam design are obtained. Out-of-plane tensile stress is calculated analytically by using experimental data. Failure modes and failure radial location of fiber-reinforced curved beams are analyzed as well. The number of curved beams per build during multiple printing is taken into account to investigate the effect of delay time between each deposited layer of parts. Macro-modelling of additively manufactured composite materials, which consider surface-based cohesive behavior, for the selected curved beam design are also presented and the numerical results are compared with the experimental results.

## 2. Materials and Methods

### 2.1. AM Method and Composite Curved Beams

A Markforged Mark Two 3D printer [32] has the capability of manufacturing continuous fiber-reinforced thermoplastics by using two heated nozzles that are fed by plastic and fiber filament spools separately. The operating temperatures are around 275 °C and 250 °C for the thermoplastic and fiber nozzles, respectively. The printer has a build volume of 320 mm (*X*; width) × 132 mm (*Y*; depth) × 154 mm (*Z*; height), which is sufficient for printing desired curved beams. The layer height (*Z* layer resolution) is achieved as 0.1 mm by reinforcing with high strength high temperature fiberglass (HSHT FG), an aramid (Kevlar^®^) and fiberglass (FG) filament as well as by using only thermoplastic substances without reinforcing. When a carbon filament is used as reinforcement, the resolution is achieved as 0.125 mm. There are some limitations to embedding fibers in the matrix. Five-roof and five-floor plastic layers are needed above and below the fiber groups in the *Z* direction. While two wall layers made of plastic are recommended to have a good surface finish and be watertight in the *XY* plane, it is mandatory to have a minimum of one wall layer that is 0.4 mm in thickness [33].

**Figure 1:**
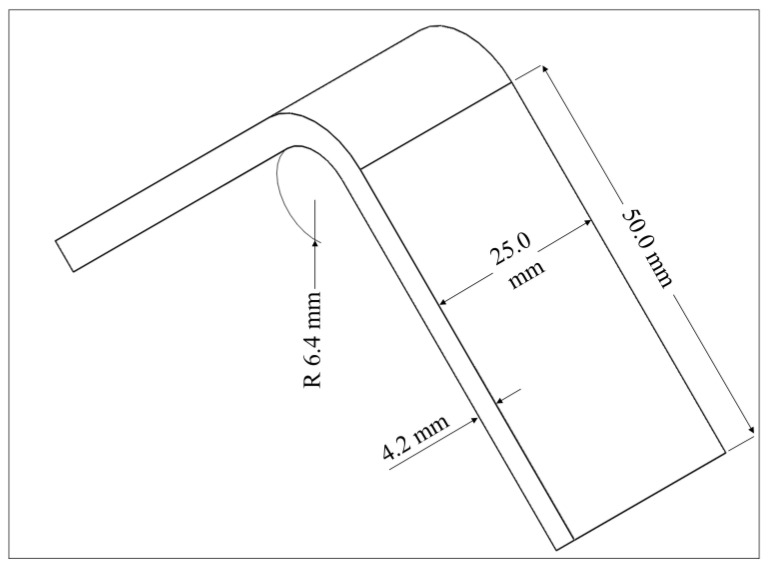
The dimensions of the curved beam design.

The design of the curved specimen is based on the ASTM D6415/D6415M-22 standard. The curved beam is designed as integrated two direct loading legs joined by a 90° curve part with a 6.4 mm inner radius (*r_i_*) while the legs and the bend are 25 mm in width (*w*) and 4.2 mm in thickness (*t*) in this study [4]. The outer radius (*r*_0_) of curved region is 10.6 mm. The length of each straight leg (*L*) is chosen as 50 mm, which is long enough to provide certain span lengths of four-point fixtures, and which is short enough to prevent contact with the fixture base. The dimensions are shown in Figure 1. A polyamide (Nylon White) is used as the thermoplastic material and is reinforced with various continuous fibers such as carbon, HSHT FG, Kevlar and FG. After drawing the L-shaped curved beam with Solidworks^®^ [34], the composite model is designed by using Eiger software [35] which enables the management of all parameters about the material and printing including the placement of fiber filaments in the matrix. After the completion of the composite design, the parts are manufactured by a Mark Two 3D printer.

**Figure 2:**
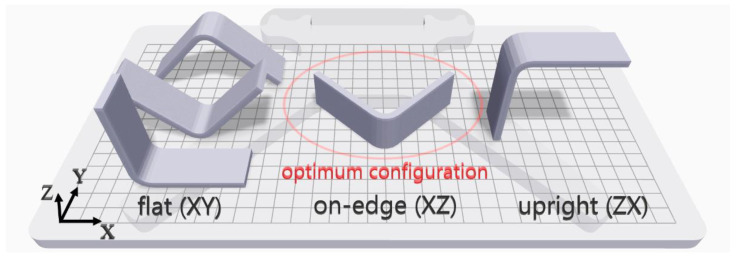
Various build orientations for printing the curved beam and the selected configuration.

Firstly, the optimum build configuration needs to be specified before the selection of other process parameters, since a curved beam is a complex structure and the print orientation affects the mechanical performance of a 3D-printed part. Three basic configurations were compared for the tensile properties of 3D-printed dog bone specimens using FDM by Chacón et al. [36] and Ma et al. [37]. The results of the flat (*XY*) and on-edge (*XZ*) orientation were measured close to each other, although the flat orientation had slightly better tensile strength. The tensile strength of the upright (*ZX*) orientation was measured at about half of the others. Figure 2 shows various build orientations in Eiger software for printing a curved beam. On-edge orientation is obviously the optimum layer-by-layer printing configuration among all possible build configurations due to the capability of having both a UD stacking sequence with the fibers running circumferentially around the curved region and a few supports for raising the part. All possible flat and upright configurations are a combination of both for the whole curved beam, since different layered regions of the beam such as each beam leg need to be built via different configurations. Building the curved beam on-edge is a pure configuration for printing, unlike the others. Moreover, each flat configuration and an upright build design need a huge amount of support material due to the curved shape. Lastly, the fiber reinforcement is not desirable and effective due to the changeable small areas of printing layers, asymmetric orientation and unbalanced fiber density between regions.

A solid fill with a density of 100% is selected as the infill pattern, which describes the repeating geometric shape inside a part. A brim is used to hold each curved beam to the print bed as the part has few points of contact on the bed that leads to warping. A raft that is made of a thick grid is also added under the beam to ensure that the warping problem is completely prevented. *t* is the most critical dimension for embedding as many continuous fibers into matrix as possible. There are two types of fiber fill algorithms, which are concentric fibers and isotropic fibers, to reinforce the part while each layer is printed in the *XY* plane. The concentric fiber type reinforces the part along any walls while the isotropic fiber type makes the part reinforced in selected directions, which are controlled by a list of angles between 0° and 180° in the *XY* plane. A concentric fiber fill that contains one fiber ring per layer (the maximum amount that is allowed by Eiger software for the selected *t*) with a start rotation percent of 0 is selected after many simulations are compared under the circumstances of the thin curved region. The number of wall layers is chosen as two since the software lets the curved beam have the same number of fiber layers in selected *t* by choosing one wall layer. The printer parameters, which are demonstrated above, are summarized in Table 1.

**Figure 3:**
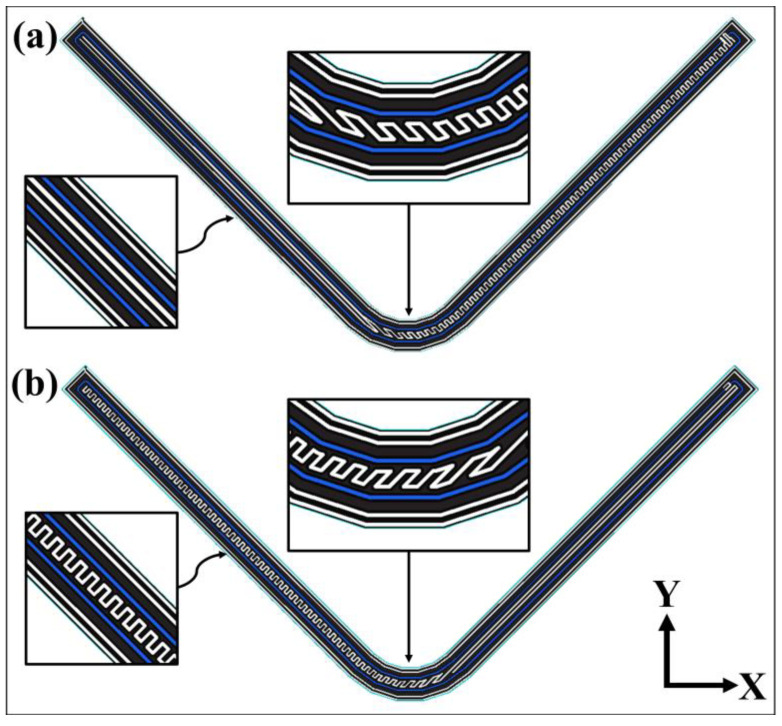
Schematic 2D view of (**a**) the initial and (**b**) the next carbon/nylon composite printing layer.

Graphical 2D views of two adjacent carbon fiber-reinforced nylon layers along the *Z* direction are shown in Figure 3 where the nozzle pattern for the fiber-reinforced lamina is represented with a blue color line. The slight difference between Figure 3a,b is the printing pattern assigned by the software for the plastic region between fiber-reinforced layers along the legs, which change symmetrically. The pair of layers is used for printing the total of fiber-reinforced layers along the *Z* direction continuously.

**Figure 4:**
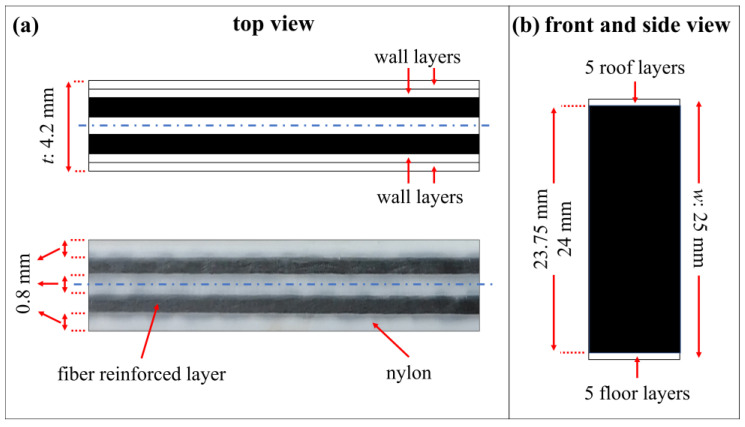
The cross-sections of (**a**) top view and the cross-section of (**b**) front and side printing views.

**Figure 5:**
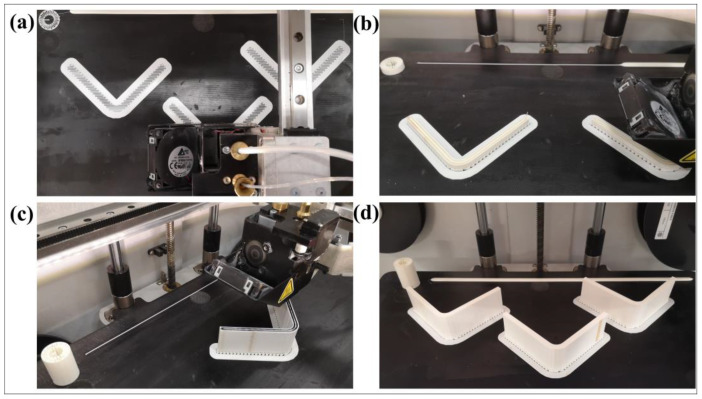
General views of the steps of manufacturing process: (**a**) printing the brim and the raft, (**b**) early-stage, (**c**) late-stage and (**d**) finished products.

The layers for HSHT FG, Kevlar and FG reinforced layers have the same printed patterns in Figure 3. However, there are more pairs of layers than a carbon/nylon specimen because of their higher *Z* layer resolution although they all have the same *w*. The carbon/nylon composite curved beams are printed by using a total of 190 fiber-reinforced layers plus a total of 10 plastic layers for the roof and the surface while the rest of the reinforced composite curved beams are printed by using a total of 240 fiber-reinforced layers plus a total of 10 plastic layers according to *Z* layer printer resolution. There is a pleasing fiber polymer laminate (FPL) design consisting of symmetrically bonded four plastic wall layers (~1.6 mm), two UD fiber-reinforced plastic lamina (~1.8 mm) and one plastic lamina (~0.8 mm) through the thickness. A cross-section of the top view, which contains fiber-reinforced layers, is depicted and the image of a carbon/nylon printing layer with the material composition is shown in Figure 4a. Moreover, a front and a side view of a curved specimen is depicted in Figure 4b. The steps of the manufacturing process, which are printing the brim and the raft, the early-stage, the late-stage and the finished products, are shown in Figure 5. The number of manufactured curved beam specimens per build is arranged as one, two and three as shown in Figure 5 to observe the effect of delay time between each layer of parts. The more specimens the printer manufactures, the more delay time the next layer has because of the layer-by-layer process. This can lead to a reduction in interlayer adhesion depending on the cooling time. Enough test coupons are printed for certain reinforcing types and each specimen is stored in a bag containing a silica gel moisture absorber until the test after printing.

The calculations of *V_f_* are made for each fiber-reinforced 3D-printed specimen by using the estimated fiber and plastic volume via Eiger software and excluding the plastic parts for the brim and raft. *V_f_* is defined in Equation (1) according to the rule of mixtures (ROM), where *υ_f_* is the volume of fiber and *υ_m_* is the volume of matrix, by neglecting the void content.

(1) $$V_{f}=\frac{\upsilon_{f}}{\upsilon_{f}+\upsilon_{m}}$$

However, the estimated *υ_f_* can be modified since the 3D printer’s fiber filaments should be defined as a kind of prepreg consisting of a bundle of long fiber-reinforced nylon. The actual diameter of the filament in the spool is measured as 0.3 mm for all fiber materials. The nylon in filament melts while it moves through the fiber nozzle and the bundle of fibers is embedded diffusely in the printing area. That is why the thickness of carbon fiber lamina looks thicker in Figure 4a. Justo et al. [38] proved that the fiber mass fraction (*W_f_*) of a carbon fiber-reinforced filament was achieved as 0.4, while the *W_f_* of an FG lamina is 0.5 after the calcination process had been carried out. HSHT FG and Kevlar filaments supposedly have the same *W_f_* as does FG. Henceforth, the estimated *V_f_* obtained in Eiger software can be described as a volumetric fiber-reinforced region (*V_r_*). It was suggested that the modified *V_f_* would be 0.4x*V_r_* for a carbon/nylon part while it would be 0.5x*V_r_* for an FG/nylon part (and so on for HSHT FG/nylon and Kevlar/nylon) by Díaz-Rodríguez et al. [39]. 

Longitudinal elastic modulus in the 0° direction (*E*_1_) and transverse elastic modulus in the 90° direction (*E*_2_), which are used in analytical functions of radial tensile stress in the unidirectional curved region, can be predicted by using the ROM and strength of materials approach in the following equations [40]:

(2) $$E_{1}=E_{f}V_{f}+E_{m}(1-V_{f})$$

(3) $$E_{2}={\left(\frac{V_{f}}{E_{f}}+\frac{\left(1-V_{f}\right)}{E_{m}}\right)}^{-1}$$

where *E_f_* and *E_m_* are elastic modulus of the fiber and matrix, respectively. The Halphin–Tsai (HT) semi-empirical model in Equation (4) is a well-known better approach for obtaining *E*_2_ since it is a fact in the literature that Equation (3) does not have a good agreement with the experimental data [40]:

(4) $$E_{2}^{HT}=E_{m}\left(\frac{1+\zeta \eta V_{f}}{1-\eta V_{f}}\right)$$

where

(5) $$\eta =\frac{\left(E_{f}/E_{m}\right)-1}{\left(E_{f}/E_{m}\right)+\zeta }$$

*ζ* is called the reinforcing factor and 2.0 is usually chosen as the default. It can be calibrated by a formula that is suggested by the HT model to be used when there are no experimental data [41]:

(6) $$\zeta =2+40V_{f}^{10}$$

In-plane material properties of a 3D-printed UD carbon and a Kevlar reinforced lamina were obtained by an experimentally validated numerical study by Galati et al. [42]. The elastic properties of FG/nylon, which were obtained by Justo et al. [38], are used in this study. The properties of HSHT FG/nylon are assumed to be the same as FG/nylon since the manufacturer’s data reported that the properties of FG and HSHT filaments have the same or close values except for flexural and compressive strengths [43]. Nylon White is a stronger and stiffer material by comparison with the conventional nylon used for 3D printers. Fisher et al. [44] obtained the elastic tension and compression properties of Nylon White at different strain rates by also considering three different infill parameters including the on-edge orientation used in this study. The material properties of the constituents used in this study are shown in Table 2.

Table 3 shows the estimated properties of each UD fiber-reinforced curved beam design. However, *V_f_* is the realistic value to estimate the amount of fiber in the curved beams, *V_r_* and the related properties in Table 2 are meaningfully used to calculate the *E*_1_, *E*_2_ and *E*_2_*^HT^* of the printed curved beams.

### 2.2. Test Method

**Figure 6:**
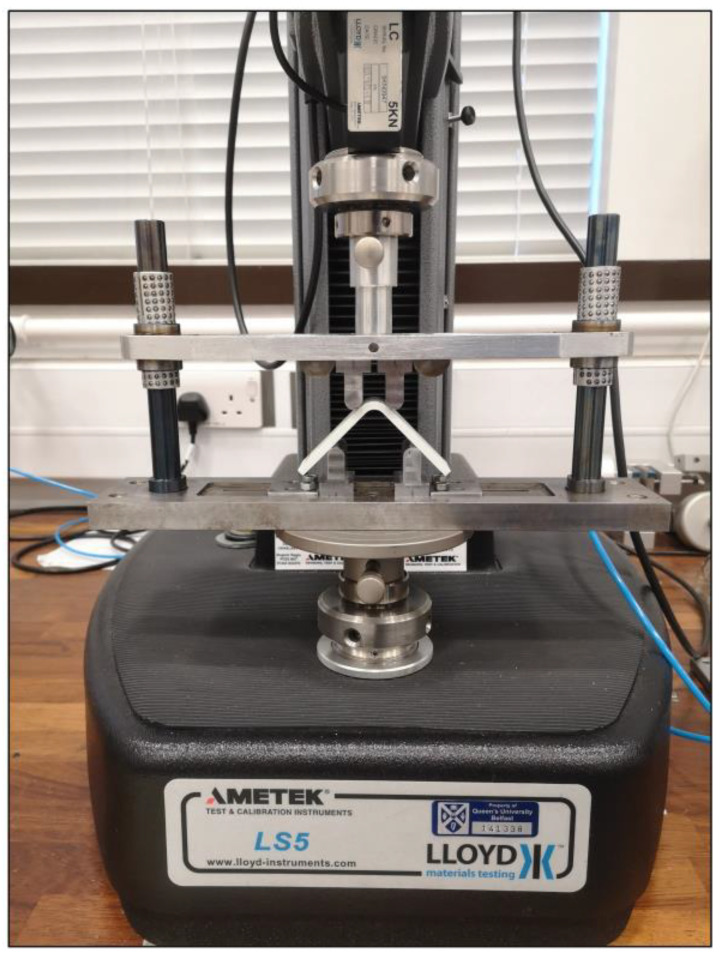
Lloyd LS5 testing machine and four-point bending apparatus.

The test procedure is based on the ASTM D6415/D6415M-22 standard. The four-point bending tests on certain types of 3D-printed composite curved beam specimens are conducted to create a sufficient moment in the bend, which produces radial tensile stress through the thickness. The tests are carried out using a Lloyd LS5 testing machine [45] and a four-point bending apparatus as shown in Figure 6. The apparatus has an upper and a lower fixture that is also integrated with two steel struts and two cylindrical ball bearings to insure against misalignment of span lengths during the head displacement.

**Figure 7:**
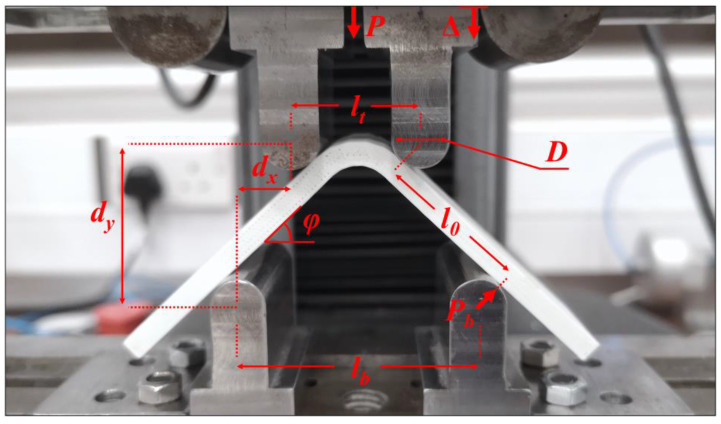
The general view of the test setup and the dimensional parameters.

The cylindrical loading and supporting steel bars have diameters (*D*) of 10 mm. The top span length (*l_t_*) is assigned as 22.1 mm to create an acceptable failure mode while the head displacement (*Δ*) is as small as it can be before damage in a bending situation. The acceptable failure mode is supposed to be a delamination in the radial position when the ILTS is obtained at the damage location. On the other hand, the distance for the bottom span length (*l_b_*) is selected as 42.1 mm to create enough moments in the curved region without excessive flexure of the specimen legs. Figure 7 shows the general view of the test setup and the parameters that configure the apparatus and test procedure as well as the analytical formulations for the strength of the curved beam. The test speed is set as 3 mm/min while the sampling rate is set as 10 Hz. Experimental load and displacement data are collected, and calculations are analytically made to obtain certain properties.

The CBS of a curved specimen is described as the ultimate applied moment per unit *w* in the curved region, which simultaneously occurs with the first force drop. A single sharp drop occurs for a UD specimen while there can be multiple force drops for a multidirectional specimen. It is a good way of measuring for the design and analysis of curved structures. The applied moment is calculated as the multiplication of the force applied to one of the supporting bars (*P_b_*) and the straight length between that supporting bar and the adjacent loading bar (*l*_0_) [4]:

(7) $$CBS=\frac{M_{ult}}{w}=\frac{P_{b}l_{0}}{w}$$

*P_b_* and *l*_0_ can be expressed in terms of the measurable parameters, which are the force applied to the fixture (*P*), angle between the specimen leg and horizontal axis (*φ*), horizontal distance between two bars along one of the legs (*d_x_*), *D* and *t*. The curved beam moment (*M*) is derived as:

(8) $$M=\left(\frac{P}{2\cos\varphi }\right)\left(\frac{d_{x}}{\cos\varphi }+\left(D+t\right)\tan\varphi \right)$$

where

(9) $$d_{x}=\frac{l_{b}-l_{t}}{2}$$

*φ* is defined as *φ_i_* when it is 45° at the start of the test. It is essential to calculate *φ* during the test since it decreases significantly as well as *d_y_* does although *d_x_* is a constant position during loading:

(10) $$\varphi =\sin^{-1}\left(\frac{-d_{x}\left(D+t\right)+d_{y}\sqrt{d_{x}^{2}+d_{y}^{2}-D^{2}-2Dt-t^{2}}}{d_{x}^{2}+d_{y}^{2}}\right)$$

where *d_y_* is vertical distance between two adjacent bars:

(11) $$d_{y}=d_{x}\tan\left(\varphi_{i}\right)+\frac{D+t}{\cos\left(\varphi_{i}\right)}-\Delta$$

ILTS is calculated by using the formula for the radial (interlaminar) tensile stress through the thickness developed by Lekhnitski [13]:

(12) $$\sigma_{r}^{\max}=-\frac{CBS}{r_{0}^{2}g}\left[1-\frac{1-\rho^{\kappa +1}}{1-\rho^{2\kappa }}{\left(\frac{r_{m}}{r_{0}}\right)}^{\kappa -1}-\frac{1-\rho^{\kappa -1}}{1-\rho^{2\kappa }}\rho^{\kappa +1}{\left(\frac{r_{0}}{r_{m}}\right)}^{\kappa +1}\right]$$

where

(13) $$\begin{array}{l} g=\frac{1-\rho^{2}}{2}-\frac{\kappa }{\kappa +1}\frac{{\left(1-\rho^{\kappa +1}\right)}^{2}}{1-\rho^{2\kappa }}+\frac{\kappa \rho^{2}}{\kappa -1}\frac{{\left(1-\rho^{\kappa -1}\right)}^{2}}{1-\rho^{2\kappa }};\\ \kappa =\sqrt{\frac{E_{\theta }}{E_{r}}};\;\rho =\frac{r_{i}}{r_{0}};\\ r_{m}={\left[\frac{\left(1-\rho^{\kappa -1}\right)\left(\kappa +1\right){\left(\rho r_{0}\right)}^{\kappa +1}}{\left(1-\rho^{\kappa +1}\right)\left(\kappa -1\right)r_{0}{}^{-\left(\kappa -1\right)}}\right]}^{\frac{1}{2\kappa }} \end{array}$$

*r_m_* is the radial position of MILTS. *E_θ_* and *E_r_* are the elastic modulus in the tangential and radial directions and should be replaced with *E*_1_ and *E*_2_ (*E*_2_*^HT^*) in Table 3 but only if a UD configuration exists. ILTS can be calculated easily in Equation (14), producing an error of less than 2% if *E_θ_*/*E_r_* (*E*_1_/*E*_2_) is less than 20 [4]. The expressions in Equations (12) and (14) do not represent a tensile stress distribution through the thickness but can show the change in MILTS during a test until having ILTS at the radial position where potential delamination occurs.

(14) $$\sigma_{r}^{\max}=\frac{3\cdot CBS}{2t\sqrt{r_{i}r_{0}}}$$

The angle between the legs, *t* and *w* of each specimen are measured before testing. The 90° angle, the 4.2 mm *t* and the 25 mm *w* are confirmed for all samples by means of the sensitive manufacturing capability of AM technology. However, the unreinforced plastic roof and floor layers in Figure 4b are neglected for the calculation of CBS and ILTS since their contribution to delamination strength is so limited by comparison with the reinforced section. *W* is consequently selected as 23.75 mm for carbon/nylon specimens and as 24 mm for the rest of the fiber reinforced specimens.

### 2.3. Finite Element Modelling

An additively manufactured curved beam subjected to four-point bending is modelled in 2D planar and 3D space using commercial Abaqus finite element software [46]. A UD carbon fiber reinforced cross-section of the beam is included in the 2D model. The deformable cross-section is created by separately modelled five layers in *t* as Figure 4a while 2D cylindrical roller, which has the specific *D*, is created as a discrete rigid wire. The laminas are first perfectly bonded in the finite element model. Table 2 is used for elastic in-plane engineering constants for a carbon/nylon lamina and White Nylon. Materials are assigned to 2D parts as sections. Material orientations in two directions are generated to form a UD laminated structure with all fibers running continuously along the curved beam. A contact property, which has both normal hard contact and frictionless tangential behavior, is created subsequently. The contact is applied between the surfaces of a 2D curved beam and the surfaces of rollers by defining a surface-to-surface contact after the assembly of a virtual test is arranged by considering *l_t_* and *l_b_*. The clamped boundary condition is defined for the lower roller while the upper roller is only allowed to have a certain displacement in the *Y* direction.

Deformable 3D UD carbon reinforced part and deformable 3D nylon part for the roof and floor of the whole curved beam, which has the same *t* and *r_i_*, are created by tying the beam in *w* as Figure 4b while the 3D cylindrical roller is created as a discrete rigid body and is then transformed to a shell body in 3D finite element model. UD carbon reinforced part consists of five layers in a *t* likewise 2D model. Out-of-plane properties in the 3-direction are assumed to be the same as the ones, which are related to the 2-direction. Materials are assigned to 3D parts as sections and material orientations in three directions are then generated. The contacts and the boundary conditions are similarly defined in the 3D model too.

**Figure 8:**
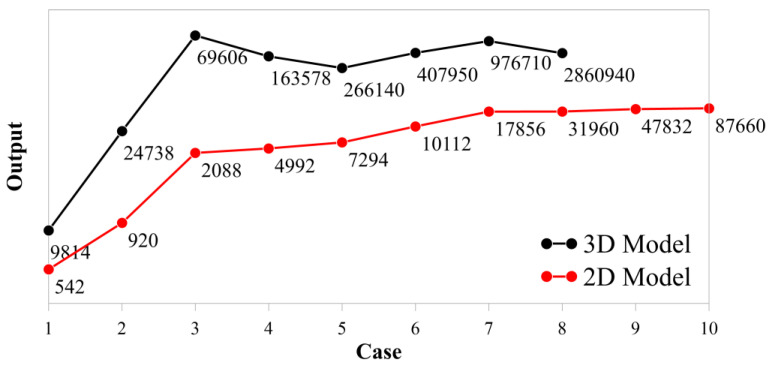
Mesh sensitivity analysis.

The 2D model of the beam is meshed using four-node plane strain elements with reduced integration (CPE4R) since the beam is thick in the *Z* direction to define a plane stress condition. The rollers are discretized using two-node 2D linear rigid elements (R2D2). On the other hand, the 3D model of the beam is discretized using eight-node 3D solid elements with reduced integration (C3D8R) while a four-node 3D bilinear quadrilateral rigid element (R3D4) is used for meshing the rollers. A general static analysis, which contains the nonlinear effects of large displacements and dissipated energy fraction for automatic stabilization, is performed for both 2D and 3D numerical modelling. The 2D and 3D full-scale models are analyzed. A mesh sensitivity analysis is first carried as shown in Figure 8. Certain cases, which have the properties in Table 4, are defined and used.

**Figure 9:**
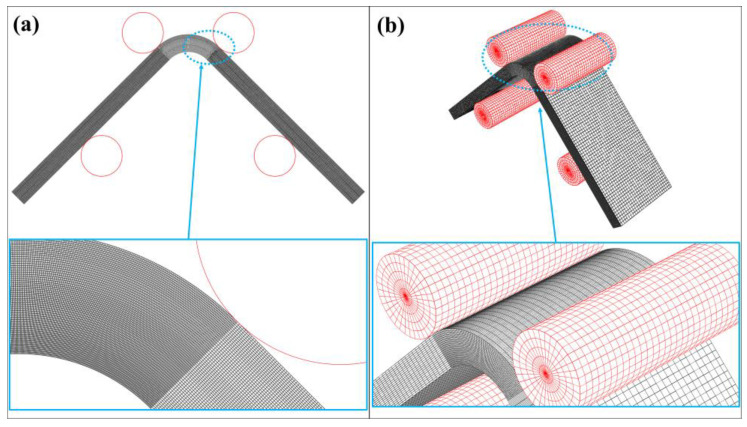
The finite element model of the virtual test by focusing on the mesh density of the curved area for (**a**) 2D and (**b**) 3D models.

The approximate global sizes for the beam and the roller model are decreased while a local mesh is applied for the critically loaded bend. The number of finite elements is increased from 10 to 350 on the curved edges of the bend and the number of elements through the beam thickness is increased from 5 to 60 by performing ten cases. The number of elements for the 2D and 3D model are also labeled for each case in Figure 8. Sufficient convergence is obtained, and the fourth case is chosen for 3D model while the last case is used for 2D model without concerning high calculation time. The finite element models of the final cases are shown by mainly focusing on the local mesh of the bend in Figure 9.

An effective elastic approach for 2D and 3D numerical models is lastly obtained by defining and applying surface-to-surface cohesive contact between certain laminas after evaluating the experimental results, especially the location of delamination and confirming that the delamination is the only damage mode. Each perfectly bonded contact in the previous models between a carbon/nylon lamina and an adjacent White Nylon lamina in Figure 4a is replaced with the bilinear traction-separation cohesive behavior. Four cohesive contacts are created between layers along the legs and the bend. The unreinforced plastic roof and floor layers are not included in the 3D numerical cohesive model.

Cohesive behavior is incorporated as a component of the surface interaction properties assigned to a contact pair rather than an inherent material property in Abaqus. These properties are input data in the linear elastic traction–separation model, criteria for initiating damage, and laws governing the evolution of damage [46]. 

The first segment of a typical traction–separation response represents the initial linear elastic behavior of the cohesive model, where the cohesive traction increases linearly with separation until reaching a critical peak traction strength point. An uncoupled model that uses penalties, which are normal (*k_nn_*) and shear stiffness components (*k_ss_*; *k_tt_*) only, is used to obtain traction stresses (*T_n_*, *T_s_*, *T_t_*) versus separation vectors (*δ_n_*, *δ_s_*, *δ_t_*):

(15) $$\left[T_{i}\right]=\left[\begin{array}{c} T_{n}\\ T_{s}\\ T_{t} \end{array}\right]=\left[k_{ii}\delta_{i}\right];\;i:n,s,t$$

Damage initiation or the degradation process in the cohesive contact at a specific point commences when the traction stresses fulfill defined specific damage initiation criteria, which is defined as quadratic stress criterion in this study:

(16) $${\left(\frac{T_{n}}{T_{n}^{0}}\right)}^{2}+{\left(\frac{T_{s}}{T_{s}^{0}}\right)}^{2}+{\left(\frac{T_{t}}{T_{t}^{0}}\right)}^{2}=1$$

where *T_n_*^0^, *T_s_*^0^ and *T_t_*^0^ represent the normal and shear strengths of cohesive contact, respectively. After the peak traction, the second linear segment of response exists where the cohesive traction decreases linearly as the separation continues to increase. A damage variable (*d*) that varies from 0 to 1 during the overall damage is used to generate the evolution model:

(17) $$\left[T_{i}\right]=(1-d)\left[k_{ii}\delta_{i}\right];\;i:n,s,t$$

An energy-based mixed mode approach based on the nature of *d* is useful to define damage evolution since the fracture energy is the area under the traction–separation curve. If *G_n_^C^*, *G_s_^C^* and *G_t_^C^*, also commonly known as *G_IC_*, *G_IIC_* and *G_IIIC_*, define the critical normal, first shear and second shear energy release rates, respectively, and *G_s_^C^* = *G_t_^C^*, Benzeggagh and Kenane (BK) fracture criterion expresses mix-mode energy dissipated due to the failure of what is defined as the function of *G_S_* (the sum of shear fracture energies) and *G_T_* (the sum of normal and shear fracture energies) where *m* is BK cohesive parameter [47]:

(18) $$G^{C}=G_{n}^{C}+\left(G_{s}^{C}-G_{n}^{C}\right){\left(\frac{G_{S}}{G_{T}}\right)}^{m}$$

Turon et al. [48] offered an equation to obtain the interface stiffness (*k*) analytically, when α is a parameter that is much bigger than 1 and each sublaminate has the thickness, *t* and out-of-plane elastic modulus, *E*_3_:

(19) $$k=\frac{\alpha E_{3}}{t}$$

*E*_3_ can be substituted for *E*_2_*^HT^* for carbon/nylon curved beam in Table 3. *t* is selected as 0.85 mm, which is the average of a fiber reinforced layer and pure nylon layer. α is defined as 50 [48]. All penalties in this study are assumed to be the same. 

Santos et al. [27] experimentally obtained *G_n_^C^* and *G_s_^C^* of 3D-printed continuous carbon fiber-reinforced polyamide while *T_s_*^0^ was investigated via short beam three-point bending tests by Yavas et al. [31]. A relation between *T_s_*^0^, *G_n_^C^* and *G_s_^C^* can define the missing cohesive contact parameter (*T_n_*^0^) in the literature [49]:

(20) $$T_{n}^{0}={\left(\frac{G_{n}^{C}}{G_{s}^{C}}\right)}^{\frac{1}{2}}T_{s}^{0}$$

The predicted interfacial properties of cohesive contact between a 3D-printed carbon/nylon layer and a nylon layer are shown in Table 5. A viscosity coefficient, which is 0.0001, is also used to regularize the convergence difficulties because of the nature of the problem in Abaqus.

## 3. Results and Discussion

### 3.1. Carbon/Nylon

**Figure 10:**
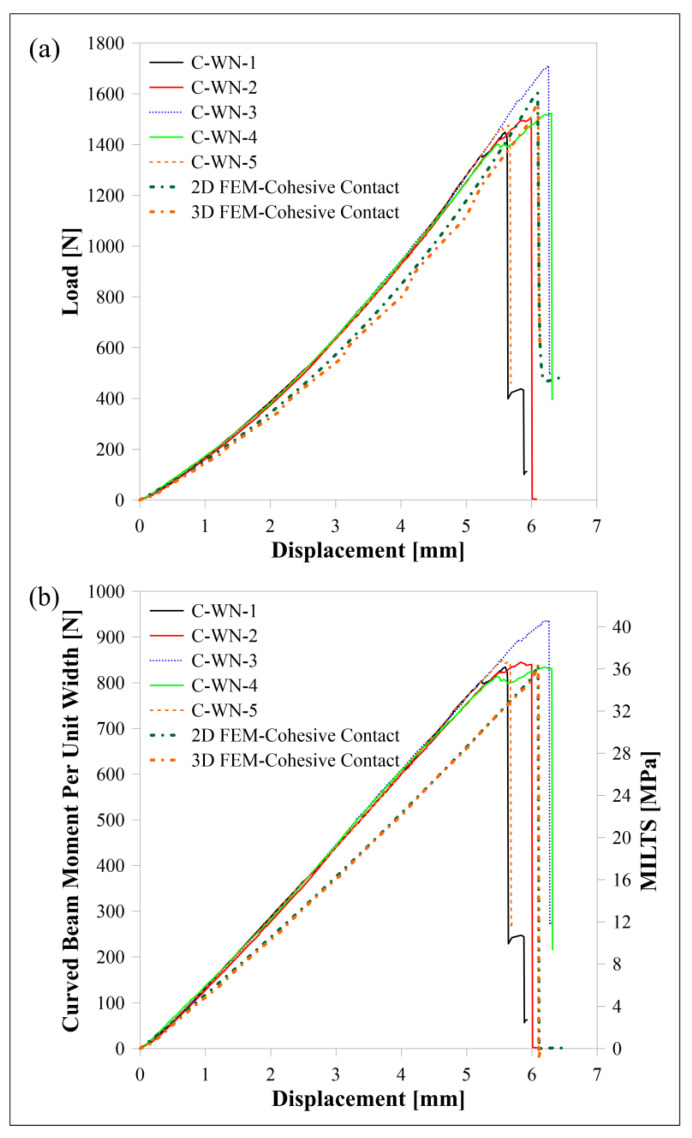
Carbon/nylon samples’ curves and FEM curves for (**a**) load displacement, (**b**) *M*/*w* displacement and MILTS displacement.

The load-displacement and moment per unit width (*M*/*w*)-displacement curves of carbon/nylon samples subjected to four-point bending are shown in Figure 10. Moreover, an additional axis is added in Figure 10b to observe the change in MILTS against the displacement until the reaching of the ILTS. The name of a sample is decoded when ‘C’ and ‘WN’ describe ‘carbon reinforcement’ and ‘Nylon White’, respectively, and the last digit describes the specimen’s number. Numerical results of 2D and 3D models with cohesive contact are also compared with experimental results in the figures. There is a good agreement between not only displacement-time histories but also MILTS-displacement histories and ILTS values at certain displacements.

**Figure 11:**
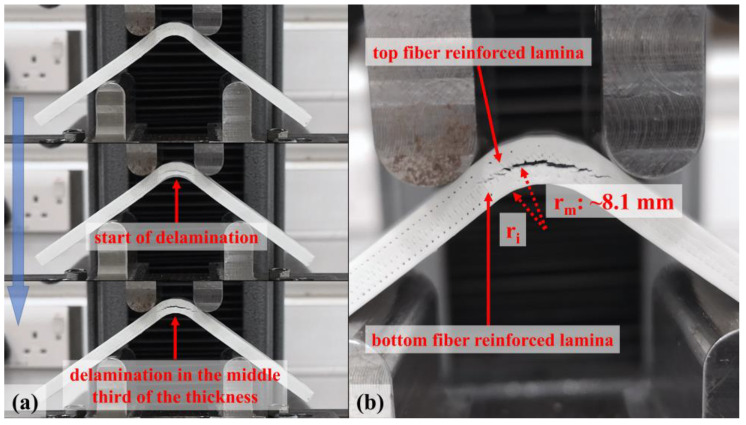
Captured images of (**a**) the growing of the delamination in a carbon/nylon composite curved beam and (**b**) radial location of the failure.

**Figure 12:**
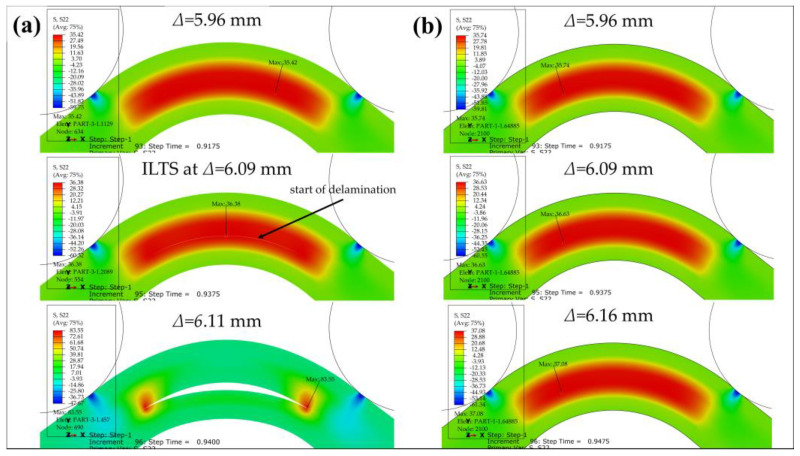
Radial tensile stress distribution of 2D (**a**) cohesive and (**b**) perfectly bonded carbon/nylon curved beam.

**Figure 13:**
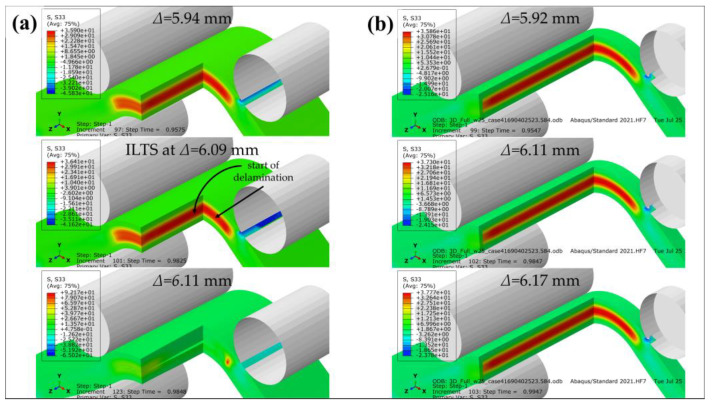
Radial tensile stress distribution of 3D (**a**) cohesive and (**b**) perfectly bonded carbon/nylon curved beam.

The curves of all test samples and numerical results have a sharp drop in force at a certain *Δ*, which is around 6 mm, as a result of the damage mechanism shown in Figure 11. The failure mode is a delamination which occurs in the contact surface between the plastic region under the mid-plane and the bottom carbon fiber-reinforced layer along the bend. A radial location of failure can be predicted as ~8.1 mm by analyzing Figure 4 and Figure 11b visually while the finite element method (FEM) verifies 8.1 mm for the location of delamination in Figure 12a and Figure 13a. On the other hand, *r_m_* values of the damaged carbon/nylon samples are calculated as 8.07 mm by using Equation (13). As a result, there is a consistency between the radial location of delamination and *r_m_*.

Radial tensile stress distributions at three different *Δ* (before damage, start of delamination and after damage) through the thickness including where ILTS exists in the bend in the surfaced-based cohesive 2D model is shown in Figure 12a and compared with the perfectly bonded 2D model’s tensile stress distributions at the same *Δ* in Figure 12b. ILTS is reported as 36.4 MPa in the middle location of the bend in the cohesive 2D model while the maximum stress is 36.6 MPa on the left side of the bend for the perfectly bonded model at *Δ*:6.09 mm. 

Figure 13 similarly shows the stress distributions of the surface-based cohesive 3D model versus the perfectly bonded 3D model through the thickness and along the width by cutting the view multiply in *Z*-plane and *X*-plane. The ILTS obtained is the same (36.4 MPa at *Δ*:6.09 mm) as the 2D model’s result since the displacements at damage are the same too and the location for start of delamination is in the middle of the 3D bend while the maximum stress is 37.3 MPa at *Δ*:6.11 mm. The delamination propagation looks faster along the width than through the thickness. The 3D perfectly bonded results contribute that the maximum radial stress similarly occurs on the left side of the bend in the cross-section while the location is close to one of bend edges along the width instead of the middle of the bend without cohesive characteristics. The effect of the unreinforced roof and floor layers on the load carrying capacity are definitely limited, as shown in Figure 13b. Therefore, they are not included in the 3D cohesive contact model to decrease the solving time.

### 3.2. HSHT FG/Nylon

**Figure 14:**
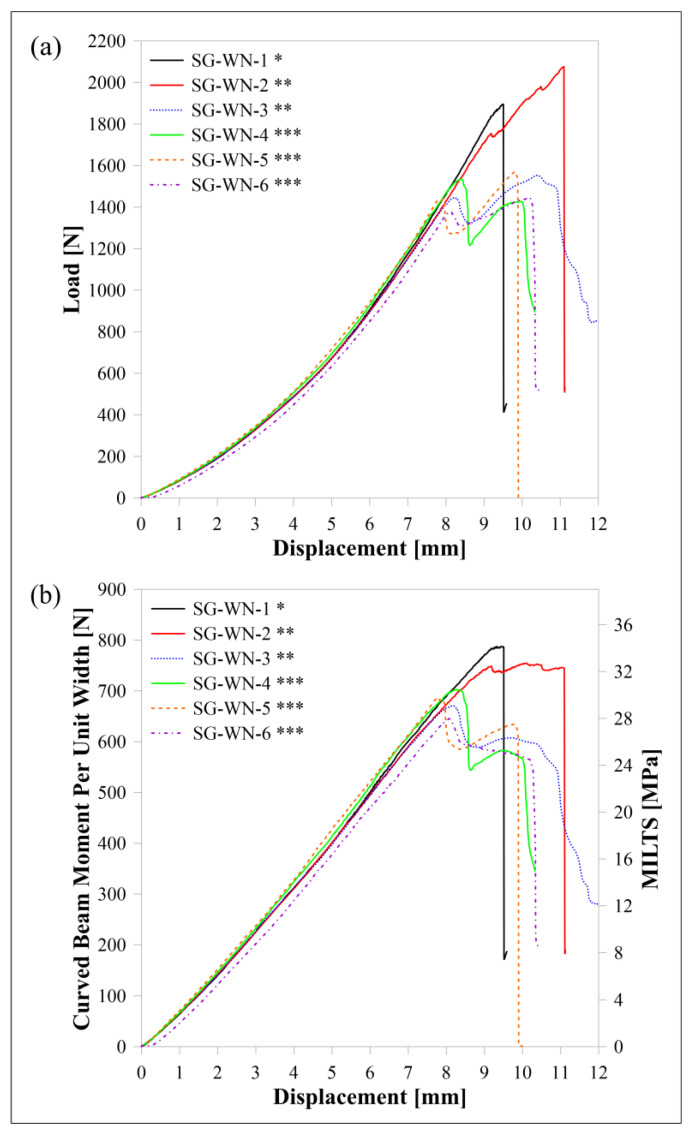
HSHT FG/nylon samples’ curves for (**a**) load displacement, (**b**) *M*/*w* displacement and MILTS displacement. * One specimen per build; ** two specimens per build; *** three specimens per build.

The experimental load-displacement, *M*/*w*-displacement and MILTS-displacement histories of HSHT FG/nylon specimens are similarly shown in Figure 14. The name of a sample is decoded when ‘SG’ describes ‘HSHT FG reinforcement’ and the other codes are ‘Nylon White’ and the specimen’s number, respectively. The symbol ‘*’ with the name of each specimen in Figure 14 refers to the number of specimens per build. It means while the specimen ‘SG-WN-1’ was printed singly, ‘SG-WN-2’ was printed with ‘SG-WN-3’ together.

The force, as well as *M*/*w*, is decreasing sharply similar to the carbon/nylon curved beam tests, although the forces of most specimens are maintained in their value around the maximum for a while. The other difference is that the failure occurs at a *Δ* of around 10 mm, which is higher than the carbon/nylon curves. The maximum loads at failure are close to, or even higher than, the carbon/nylon ones. However, CBS and ILTS are obviously lower because of reaching a smaller *φ* and *l*_0_ and having a lower force at a certain *Δ,* which is a sign of lower bending stiffness. This information also shows that observing load and a sudden drop in force is an effective method for understanding failure. However, comparing the performance of a curved beam in terms of the maximum load instead of CBS or ILTS creates a misleading impression in a study as well as in the literature. There is no distinct effect of delay time between each deposited layer of parts on the strength for double and triple printing while the single sample per build has a higher CBS and ILTS.

**Figure 15:**
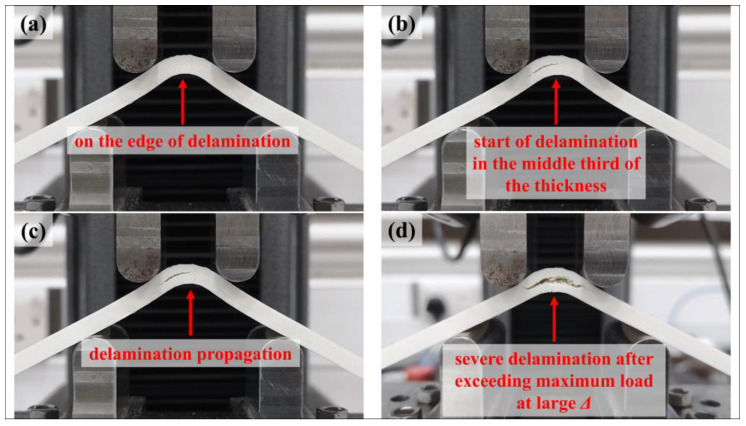
The growing of the delamination of a HSHT FG/nylon composite curved beam: (**a**) before starting; (**b**) start of delamination; (**c**) propagation; (**d**) severe delamination after exceeding maximum load at a large *Δ*.

Figure 15 shows the captured images of an HSHT FG/nylon composite curved beam (a) just before the starting of failure and (b-c-d) the images of how the delamination propagates in the middle third of the thickness. The approximate radial location of delamination is detected as ~8.1 mm in the same contact surface too after detailed visual control. The calculated *r_m_* value of the HSHT FG/nylon samples is 8.07 mm. The failure mode of the HSHT FG/nylon curved beam is acceptable as a carbon/nylon curved beam.

### 3.3. FG/Nylon and Kevlar/Nylon

**Figure 16:**
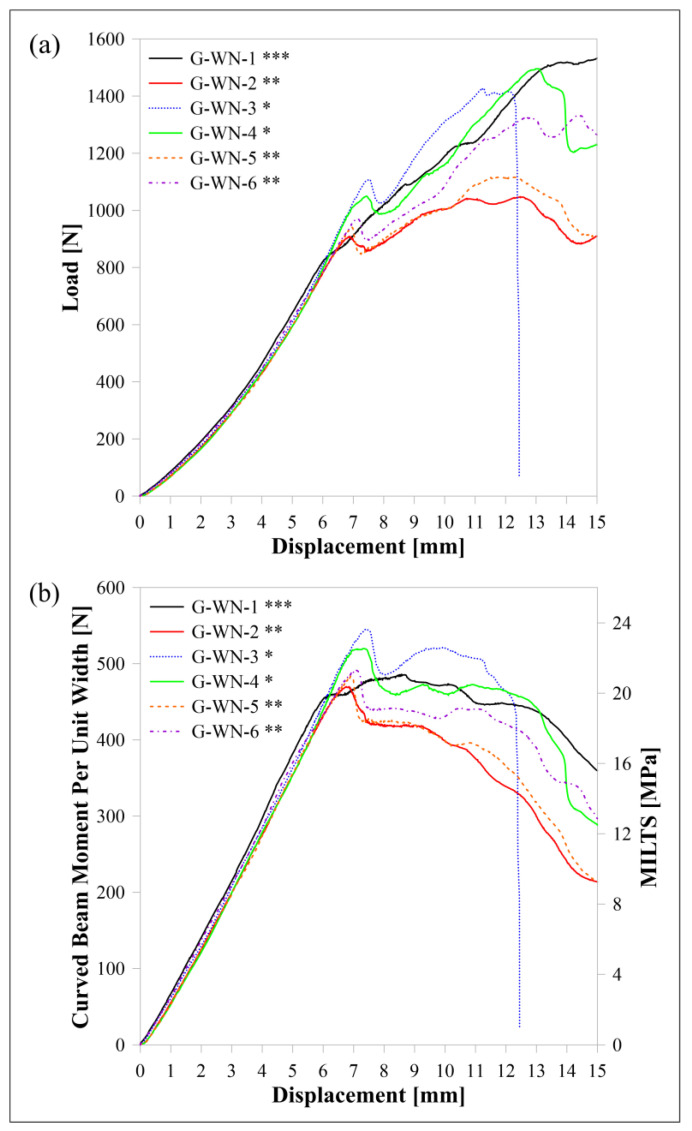
FG/nylon samples’ curves for (**a**) load displacement, (**b**) *M*/*w* displacement and MILTS displacement. * One specimen per build; ** two specimens per build; *** three specimens per build.

The load-displacement, *M*/*w*-displacement and MILTS-displacement curves of FG/nylon curved beams are shown in Figure 16. ‘G’ refers to ‘FG reinforcement’ while the other codes are the same as carbon and HSHT FG reinforced samples. A test is stopped if *Δ* reaches 15 mm before failure as *φ* is so small (less than 5° after 15 mm) to define the beam as a curved structure. No sharp drop in force or multiple force drops until 50% of the maximum load are observed for FG/nylon samples except ‘G-WN-3’ although all samples have a maximum load. CBS and ILTS are calculated and shown for comparison. The number of specimens per build is analyzed for the FG/nylon curved beam too. There is no distinct effect of delay time between each deposited layer of parts on the strength for double and triple printing while the samples, which are printed singly (‘G-WN-3’ and ‘G-WN-4’), have a higher CBS and ILTS. The specimen which has a sudden decrease in load is one of them.

**Figure 17:**
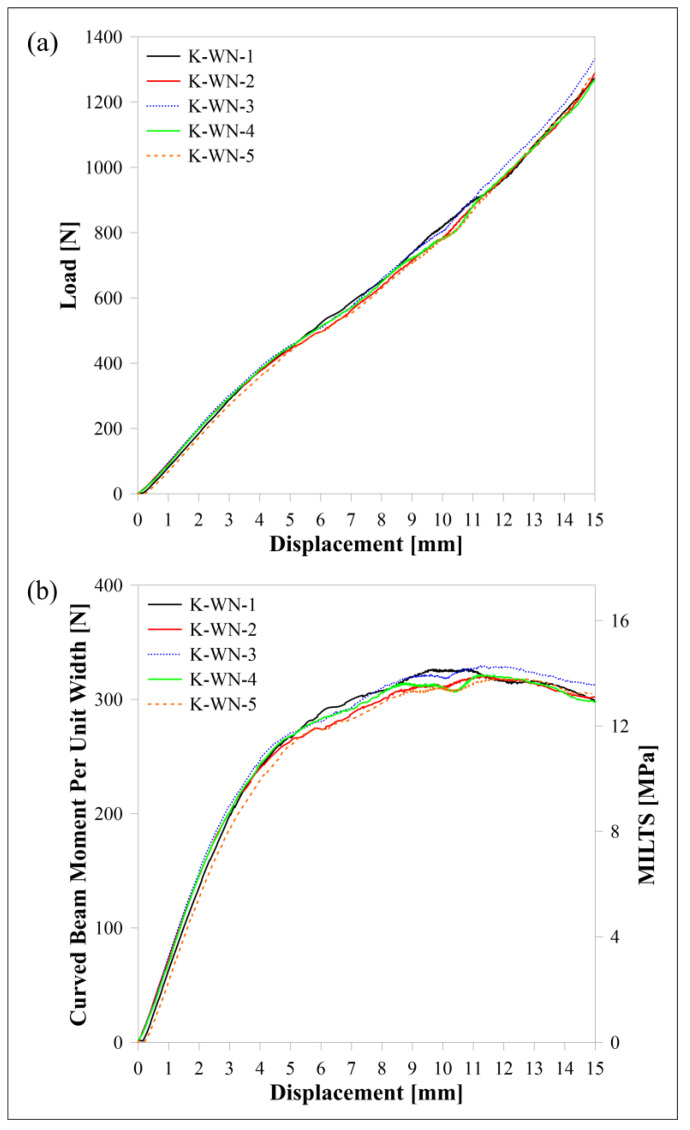
Kevlar/nylon samples’ curves for (**a**) load displacement, (**b**) *M*/*w* displacement and MILTS displacement.

**Figure 18:**
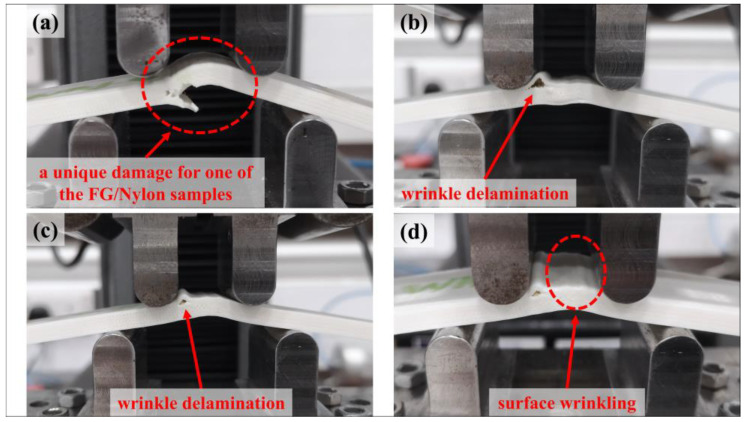
A unique damage for (**a**) an FG/nylon sample; wrinkle delamination on (**b**) a Kevlar/nylon and (**c**) an FG/nylon sample; (**d**) a surface wrinkling on Kevlar/nylon sample.

The curves of Kevlar/nylon composite curved beams are shown in Figure 17. ‘K’ refers to ‘Kevlar reinforcement’ and the other codes are defined the same as other types of reinforced samples. All tests are stopped when *Δ* reaches 15 mm and neither a sudden drop nor small decrease in force is observed during the tests although the CBS and ILTS values are obtained for comparison. It is clear that the specimen legs of the designed FG/nylon and Kevlar/nylon curved beams display extreme bending as a result of their low bending stiffness, which is the lowest for the Kevlar/nylon curved beam. Figure 18 shows typical failure modes of FG/nylon and Kevlar/nylon composite curved beams. The sample in Figure 18a is ‘G-WN-3’, which has a sudden drop in force, and has unacceptable delamination because of the radial position. The rest of all FG/nylon and Kevlar/nylon samples have failure modes in Figure 18b–d. These failure modes occur as a wrinkle delamination or surface wrinkling of the top wall layers during the loading and there is no significant effect on the change in load for Kevlar/nylon samples. It can be the reason for damage at high *Δ* and low *φ* values as well as the location of the failure which occurs at the upper surface of the outmost plastic wall layer.

### 3.4. Discussion of Results

The maximum load, CBS, ILTS via Equation (12) and ILTS via Equation (14) of all specimens are summarized and the mean values with standard deviations (SDs) are compared for each curved beam design in Table 6. The difference between the specimens’ ILTS and ILTS^Equation (14)^ is less than 0.5% for certain curved beams while both values are the same for carbon/nylon results. The carbon/nylon curved beam design has the maximum value for ILTS, which is 37.3 MPa while the strength of HSHT FG/nylon beam is 6.6 MPa lower approximately. The ILTS of FG/nylon and Kevlar/nylon beams are 21.6 MPa and 14.0 MPa, respectively; however, it must be kept in mind that they have undesirable failure modes. The maximum loads of Kevlar/nylon samples are defined as not available because of no sign of force drop within the limit of desired test *Δ*. 

The positive effect of the single specimen per build on the CBS and ILTS is shown in Table 6. While the ILTS of a single HSHT FG/nylon specimen per build is 34.1 MPa, the mean ILTS of double and triple specimens per build are 30.9 MPa and 29.4 MPa, respectively. Similarly, the mean ILTS of single and double FG/nylon specimens per build is 23.1 MPa and 20.8 MPa, respectively, while the ILTS of the specimen, which was printed together with the other two specimens, is 21.0 MPa.

The ILTS values of various carbon reinforced composite curved beam examples, which were manufactured by any conventional methods in the literature, are categorized in Table 7 to demonstrate the competitiveness of an additively manufactured composite curved beam. Comparing with UD examples is more meaningful because of having a similar lay-up. Although ILTS of 3D-printed UD carbon/nylon curved beam in this study is lower than the UD ones in Table 7 as expected, the strength value is close to some of the examples surprisingly when *V_f_* of a prepreg and 3D-printed composite are regarded. On the other hand, ILTS looks higher than the other advanced carbon reinforced application that has an innovation such as reinforcement with carbon nanotube or carbon-reinforced ceramic materials. When the performance is compared with a real aerospace bracket material [8], the necessary ILTS is decreased by around 27%.

Table 8 discusses the comparison of numerical results with the experimental results of the carbon/nylon curved beam design via a percentage difference (PD). The cohesive surface-based models are more realistic and the PD values also show there are really good agreements with experimental results for all properties especially in a 3D cohesive model. The *Δ* of perfectly bonded models depends on cohesive models and is selected as the same or an available close value. Load and stress values and *r_m_*s are then generated for perfectly bonded models to see the effect of contact type.

**Figure 19:**
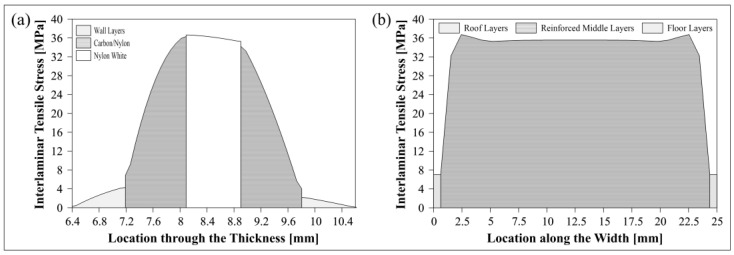
Change in the interlaminar tensile stress in the paths (**a**) through the thickness and (**b**) along the width of the bend.

Figure 19 emphasizes the changes in the interlaminar tensile stress (a) in a path of nodes through the thickness in the perfectly bonded 2D model where the maximum stress is obtained at Δ:6.09 in Figure 12b and (b) in the middle path along the width of the half-cut bend in the perfectly bonded 3D model. Figure 19a is generated for comparing the contributions of each layer to the energy absorption capacity through the thickness while Figure 19b evaluates the limited contribution of plastic roof-floor layers to strength when it is compared with the main reinforced parts in width. The maximum stress along the width is obtained at around 2.5 mm away from each edge of the bend. Stress value decreases by around 3% and stabilizes after reaching the maximum value along the width.

## 4. Conclusions

This study contributes to the raised awareness of the research of additively manufactured simplified models of composite brackets, which has not been studied in detail before. The availability of four-point bending test method of a composite curved beam, which has been limited to use molding-based conventionally manufactured composites, is also confirmed for CFF-based composites if their bending stiffness is high enough. The 3D-printed continuous fiber-reinforced thermoplastic curved beams, which are made of several types of fibers, subjected to four-point bending tests are discussed experimentally to this end. 

Although *l_t_* is kept as short as possible, the results for delamination strengths of carbon/nylon and HSHT FG/nylon curved beams are found to be acceptable since their failure modes are observed as delamination. The legs of Kevlar and FG samples exhibit excessive bending during loading without delamination due to their low bending stiffness. ILTS of carbon/nylon curved beam looks quite competitive with some of the similar UD curved beam examples in the literature while the strength of HSHT FG/nylon beam decreases by 18%. The strengths of FG/nylon and Kevlar/nylon beams are not challenging against the carbon/nylon curved beam due to not only a decrease of 42% and 62%, respectively, but also the unacceptable failure modes and loading behaviors. 

The observed radial damage locations of delamination for carbon/nylon and HSHT FG/nylon are also consistent with the analytically calculated *r_m_* values. There is no distinct effect of delay time between each deposited layer of parts on the strength for double and triple printing while printing a single sample is favorable for better strength. 

The experimental and analytical results are compared with the ones obtained by using the commercial finite element software Simulia Abaqus/CAE 2021 and are found to be in a good agreement. Surface-based cohesive models exhibit more realistic behaviors than perfectly bonded models as expected, although the cohesive behavior is modelled as a zero-thickness cohesive contact instead of cohesive elements which have cohesive material properties with finite thickness. On the other hand, the perfectly bonded model is especially effective for sensitivity analysis since it has a low CPU time and cohesive models have converging problems after delamination starts that leads to massive CPU time. The 2D model is a fast solution for obtaining a good agreement with test samples’ results when the 2D and 3D cohesive models are compared. Converging problems in cohesive models are definitely much more common for the 3D cohesive model.

For future work, it would be interesting to investigate the effects of thickness and width as well as create a hybrid composite by changing the matrix material to short carbon fiber reinforced nylon, especially for FG and Kevlar reinforced curved beams, on the delamination strength as a parametric study experimentally and numerically.

## Figures and Tables

**Table 1:** The chosen printer parameters.

Parameter	Selected Option
Fill Pattern	Solid Fill
Fill Density	100%
Wall Layers	2 (0.8 mm)
Fiber Pattern Type	Entire Group
Fiber Fill Type	Concentric Fiber
Concentric Fiber Rings	1
Start Rotation Percent	0

**Table 2:** Material properties of the constituents.

**Material**	***E*_1_ [GPa]**	***E*_2_ [GPa]**	***ν*_12_**	***G*_12_ [GPa]**
Carbon/Nylon [42]	35.00	7.50	0.15	4.00
HSHT FG/Nylon	25.86	1.13	0.37	0.88
FG/Nylon [38]	25.86	1.13	0.37	0.88
Kevlar/Nylon [42]	31.00	1.50	0.20	0.70
**Material**	***E* [GPa]**		***ν***	
Nylon White [44]	1.53		0.39	

**Table 3:** Predicted properties of the entire curved beams.

		Carbon/Nylon Curved Beam	HSHT FG/Nylon Curved Beam	Kevlar/Nylon Curved Beam	FG/Nylon Curved Beam
*υ_m_*	cm^3^	11.04	11.00	11.00	11.00
*υ_f_*	cm^3^	4.83	4.89	4.89	4.89
*V_f_*	%	12.20	15.40	15.40	15.40
*V_r_*	%	30.40	30.80	30.80	30.80
*E*_1_	GPa	11.70	9.02	10.61	9.02
*E*_2_	GPa	2.02	1.38	1.52	1.38
*E*_2_ *^HT^*	GPa	2.48	1.40	1.52	1.40

**Table 4:** Case properties of mesh sensitivity analysis.

Case	Approximate Global Size		Local Mesh
	Curved Beam	Roller	
1	2.5	1.4	10 × 5
2	1.4	1.4	20 × 5
3	1.0	1.0	40 × 10
4	0.9	0.9	90 × 20
5	0.8	0.8	120 × 25
6	0.7	0.7	150 × 30
7	0.5	0.5	200 × 40
8	0.3	0.3	250 × 50
9	0.2	0.2	300 × 55
10	0.1	0.1	350 × 60

**Table 5:** Predicted interaction properties of cohesive contact.

*k_nn_ = k_ss_* = *k_tt_* ^(Equation (19))^	MPa/mm	146,000
*G_n_^C^* [27]	mJ/mm^2^	1.7
*G_s_^C^* = *G_t_^C^* [27]	mJ/mm^2^	2.3
*m*		2
*T_n_*^0^ ^(Equation (20))^	MPa	35.2
*T_s_*^0^ = *T_t_*^0^ [31]	MPa	40.9

**Table 6:** The comparative results of certain types of composite curved beams.

**Carbon/Nylon**	**Maximum Load [N]**	**CBS [N]**	**ILTS [MPa]**	**ILTS^(Equation (14))^ [MPa]**
C-WN-1	1448	834.1	36.2	36.2
C-WN-2	1504	845.0	36.6	36.6
C-WN-3	1711	936.8	40.6	40.6
C-WN-4	1523	834.3	36.2	36.2
C-WN-5	1476	848.6	36.8	36.8
Mean (SD)	1532 (93)	859.8 (38.9)	37.3 (1.7)	37.3 (1.7)
**HSHT FG/Nylon**	**Maximum Load [N]**	**CBS [N]**	**ILTS [MPa]**	**ILTS^(Equation (14))^ [MPa]**
SG-WN-1 *	1896	787.7	34.1	34.2
SG-WN-2 **	2076	754.7	32.7	32.7
SG-WN-3 **	1553	670.5	29.0	29.1
SG-WN-4 ***	1534	702.8	30.4	30.5
SG-WN-5 ***	1571	687.9	29.8	29.8
SG-WN-6 ***	1448	645.2	27.9	28.0
Mean (SD)	1680 (226)	708.1 (48.8)	30.7 (2.1)	30.7 (2.1)
**FG/Nylon**	**Maximum Load [N]**	**CBS [N]**	**ILTS [MPa]**	**ILTS^(Equation (14))^ [MPa]**
G-WN-1 ***	1533	486.0	21.0	21.1
G-WN-2 **	1048	469.8	20.3	20.4
G-WN-3 *	1428	545.5	23.6	23.7
G-WN-4 *	1496	519.8	22.5	22.5
G-WN-5 **	1116	483.5	20.9	21.0
G-WN-6 **	1333	492.4	21.3	21.4
Mean (SD)	1326 (184)	499.5 (25.5)	21.6 (1.1)	21.7 (1.1)
**Kevlar/Nylon**	**Maximum Load [N]**	**CBS [N]**	**ILTS [MPa]**	**ILTS^(Equation (14))^ [MPa]**
K-WN-1	n/a	326.7	14.1	14.2
K-WN-2	n/a	320.7	13.9	13.9
K-WN-3	n/a	329.4	14.3	14.3
K-WN-4	n/a	321.5	13.9	13.9
K-WN-5	n/a	317.8	13.7	13.8
Mean (SD)	n/a	323.2 (4.22)	14.0 (0.2)	14.0 (0.2)

**Table 7:** ILTS values of various composite curved beam examples in the previous studies.

Ref.	Material System	*t* [mm]	*l_t_* [mm]	*l_b_* [mm]	ILTS [MPa]
	Present Study	4.2	22.1	42.1	37
[16]	Eight UD carbon/epoxy plies	6.7	75	100	44
[50]	T700GC/M21 UD carbon/epoxy prepreg plies	4.2	30	66	44
		12.6	53	79	37
[51]	36 IM7/8552 UD carbon/epoxy prepreg plies	6.6	n/a	n/a	73
[52]	[0_4_/90/0_3_/90/0_2_/90_2_/0_2_/90]_S_ 32 AS4/3501-6 prepreg plies	5.8	75	100	48
[19]	[0/90] HexPly^®^ AS4 5HS carbon/epoxy prepreg plies	3	n/a	n/a	28
[8]	A hybrid multidirectional aerospace application	2.5	21.7	41.9	51
[19]	[0/90] HexPly^®^ AS4 5HS carbon/epoxy prepreg plies + 3% wt carbon nanotube	3	n/a	n/a	10
[53]	2D plain woven C/C–SiC	10	75	100	19

**Table 8:** Comparison of numerical results of carbon/nylon curved beam with experimental ones.

	Maximum Load [N]	ILTS [MPa]	*Δ* [mm]	*r_m_* [mm]
Means of C-WN Specimen Series	1532	37.3	6.00	8.07
2D FEM-Cohesive Contact	1604	36.4	6.09	8.1
2D FEM PD (%)	4.7	2.4	1.5	0.4
3D FEM-Cohesive Contact	1560	36.4	6.09	8.1
3D FEM PD (%)	1.8	2.4	1.5	0.4
2D FEM-Perfectly Bonded	1616	36.6	6.09	8.1
2D FEM PD (%)	5.5	1.7	n/a	0.4
3D FEM-Perfectly Bonded	1659	37.3	6.11	8.3
3D FEM PD (%)	8.3	0.05	n/a	2.9

## Data Availability

The data presented in this study are available on request from the corresponding author.

## References

[1] Materials and Processes: Fabrication Methods. https://www.compositesworld.com/articles/fabrication-methods.

[2] 3D Printing Industry–Worldwide Market Size 2020–2026. https://www.statista.com/statistics/315386/global-market-for-3d-printers/#:~:text=The%20worldwide%20market%20for%203D,percent%20between%202022%20and%202024.

[3] Handwerker M., Wellnitz J., Marzbani H. (2021). Review of mechanical properties of and optimisation methods for continuous fibre-reinforced thermoplastic parts manufactured by fused deposition modelling. Prog. Addit. Manuf..

[4] ASTM D6415/D6415M-22 Standard Test Method for Measuring the Curved Beam Strength of a Fiber-Reinforced Polymer-Matrix Composite. https://www.astm.org/d6415_d6415m-22.html.

[5] Zou X., Yan S., Matveev M., Rouse J.P., Jones I.A. (2021). Experimental and numerical investigation of interface damage in composite L-angle sections under four-point bending. J. Compos. Mater..

[6] Wu T., Zhou G., Cai D., Zhou F., Cai L. (2021). Effect of internal heating on delamination properties of deicing composite curved beams under four-point bending. Compos. Struct..

[7] Qian S., Liu X., Ye Y., Xu Q., Zhang T., Li X. (2021). Effect of gap and overlap fiber placement defects on the delamination behavior of L-shaped composite laminates. Compos. Struct..

[8] Luinge H., Warnet L.L. (2020). On an application of multi-material composite laminates in the aerospace sector. Adv. Compos. Hybrid Mater..

[9] Ranz D., Cuartero J., Castejon L., Miralbes R., Valladares D. (2022). Enhanced cohesive zone model to predict delamination behavior of carbon/epoxy laminated curved beams. Mech. Adv. Mater. Struct..

[10] Xu X., Jones M.I., Ali H., Wisnom M.R., Hallett S.R. (2020). Effect of out-of-plane wrinkles in curved multi-directional carbon/epoxy laminates. Compos. Sci. Technol..

[11] Woo K., Nega B.F., Cairns D.S., Lua J. (2020). Delamination behavior of L-shaped composite beam with manufacturing defects. J. Mech. Sci. Technol..

[12] Petersen E., Kappel E., Koord J., Völkerink O., Hühne C. (2020). Determination of stresses, strains and failure types in multidirectional laminates under pure bending. J. Compos. Mater..

[13] Lekhnitskii S.G. (1968). Anisotropic Plates.

[14] Ranz D., Cuartero J., Castejon L., Miralbes R. (2020). A study on interlaminar behavior of carbon/epoxy laminated curved beams by use of acoustic emission. Mech. Adv. Mater. Struct..

[15] Arki S., Ferrero J.F., Marguet S., Redonnet J.M., Aury A. (2019). Strengthening of a curved composite beam by introducing a flat portion. Compos. Struct..

[16] Ranz D., Cuartero J., Miravete A., Miralbes R. (2017). Experimental research into interlaminar tensile strength of carbon/epoxy laminated curved beams. Compos. Struct..

[17] Ju H., Nguyen K.H., Chae S.S., Kweon J.H. (2017). Delamination strength of composite curved beams reinforced by grooved stainless-steel z-pins. Compos. Struct..

[18] Fletcher T.A., Kim T., Dodwell T.J., Butler R., Scheichl R., Newley R. (2016). Resin treatment of free edges to aid certification of through thickness laminate strength. Compos. Struct..

[19] Arca M.A., Uyar I., Coker D., Tandon G. (2015). Experimental investigation of the effect of CNT addition on the strength of CFRP curved composite beams. Composite, Hybrid, and Multifunctional Materials Volume 4.

[20] Hao W., Yuan Y., Zhu J., Chen L. (2016). Effect of impact damage on the curved beam interlaminar strength of carbon/epoxy laminates. J. Adhes. Sci. Technol..

[21] Fijul Kabir S.M., Mathur K., Seyam A.F.M. (2020). A critical review on 3D printed continuous fiber-reinforced composites: History, mechanism, materials and properties. Compos. Struct..

[22] Eren Z., Burnett C.A., Wright D., Kazancı Z. (2023). Compressive characterisation of 3D printed composite materials using continuous fibre fabrication. Int. J. Lightweight Mater. Manuf..

[23] Damodaran V., Castellanos A.G., Milostan M., Prabhakar P. (2018). Improving the mode-II interlaminar fracture toughness of polymeric matrix composites through additive manufacturing. Mater. Des..

[24] Touchard F., Arnault L.C., Fournier T., Magro C., Lafitte A., Caradec A. (2021). Interfacial adhesion quality in 3D printed continuous CF/PA6 composites at filament/matrix and interlaminar scales. Compos. Part B Eng..

[25] Gonzalez C.P., San Martin P., Lizarralde I., Fernandez A., Leon A., Lopes C.S., Fernandez-Blazques J.P. (2021). Post-processing effects on microstruture, interlaminar and thermal properties of 3D printed continous carbon fibre composites. Compos. Part B Eng..

[26] Santos J.D., Guerrero J.M., Blanco N., Fajardo J.I., Paltan C.A. (2023). Numerical and experimental analysis of the mode I interlaminar fracture toughness in multidirectional 3D-printed thermoplastic composites reinforced with continuous carbon fiber. Polymers.

[27] Santos J.D., Fernández A., Ripoll L., Blanco N. (2022). Experimental characterization and analysis of the in-plane elastic properties and interlaminar fracture toughness of a 3D-printed continuous carbon fiber-reinforced composite. Polymers.

[28] Yang C., Tian X., Liu T., Cao Y., Li D. (2017). 3D printing for continuous fiber reinforced thermoplastic composites: Mechanism and performance. Rapid Prototyp. J..

[29] Caminero M.A., Chacon J.M., Garcia-Moreno I., Reverte J.M. (2018). Interlaminar bonding performance of 3D printed continuous fibre reinforced thermoplastic composites using fused deposition modelling. Polym. Test..

[30] Iragi M., Pascual-Gonzalez C., Esnaola A., Lopes C.S., Aretxabaleta L. (2019). Ply and interlaminar behaviours of 3D printed continuous carbon fibre-reinforced thermoplastic laminates; effects of processing conditions and microstructure. Addit. Manuf..

[31] Yavas D., Zhang Z., Liu Q., Wu D. (2021). Interlaminar shear behavior of continuous and short carbon fiber reinforced polymer composites fabricated by additive manufacturing. Compos. Part B Eng..

[32] Markforged Mark Two 3D Printer. https://markforged.com/3d-printers/mark-two.

[33] Markforged Design Guide for 3D Printing with Composites. https://static.markforged.com/downloads/CompositesDesignGuide.pdf.

[34] Dassault Systemes Solidworks 2021 Software. https://www.solidworks.com/.

[35] Markforged Eiger Software. https://www.eiger.io/.

[36] Chacón J.M., Caminero M.A., Garcia-Plaza E., Nunez P.J. (2017). Additive manufacturing of PLA structures using fused deposition modelling: Effect of process parameters on mechanical properties and their optimal selection. Mater. Des..

[37] Ma C., Faust J., Roy-Mayhew J.D. (2021). Drivers of mechanical performance variance in 3D-printed fused filament fabrication parts: An onyx FR case study. Polym. Compos..

[38] Justo J., Távara L., García-Guzmán L., París F. (2018). Characterization of 3D printed long fibre reinforced composites. Compos. Struct..

[39] Díaz-Rodríguez J.G., Pertúz-Comas A.D., González-Estrada O.A. (2021). Mechanical properties for long fibre reinforced fused deposition manufactured composites. Compos. Part B Eng..

[40] Kaw A.K. (2005). Mechanics of Composite Materials.

[41] Vignoli L.L., Savi M.A., Pacheco P.M.C.L., Kalamkarov A.L. (2019). Comparative analysis of micromechanical models for the elastic composite laminae. Compos. Part B Eng..

[42] Galati M., Viccica M., Minetola P. (2021). A finite element approach for the prediction of the mechanical behaviour of layered composite produced by continuous filament fabrication (CFF). Polym. Test..

[43] Markforged Material Data Sheet Composites. https://www-objects.markforged.com/craft/materials/CompositesV5.2.pdf.

[44] Fisher T., Almedia J.H.S., Falzon B.G., Kazancı Z. (2023). Tension and compression properties of 3D-printed composites: Print orientation and strain rate effects. Polymers.

[45] Lloyd Instruments Lloyd LS5 Testing Machine. https://www.ametektest.com/products/material-testers/single-column-test-stands/ls-series.

[46] Simulia Abaqus/CAE 2021 Software. https://www.3ds.com/products-services/simulia/products/abaqus/.

[47] Benzeggagh M.L., Kenane M. (1996). Measurement of mixed-mode delamination fracture toughness of unidirectional glass/epoxy composites with mixed-mode bending apparatus. Compos. Sci. Technol..

[48] Turon A., Davila C.G., Camanho P.P., Costa J. (2007). An engineering solution for mesh size effects in the simulation of delamination using cohesive zone models. Eng. Fract. Mech..

[49] Song K., Davila J.F., Rose C. Guidelines and parameter selection for the simulation of progressive delamination. Proceedings of the ABAQUS User’s Conference.

[50] Charrier J.S., Laurin F., Carrere N., Mahdi S. (2016). Determination of the out-of-plane tensile strength using four-point bending tests on laminated L-angle specimens with different stacking sequences and total thicknesses. Compos. Part B Eng..

[51] Makeev A., Seon G., Nikishkov Y., Lee E. (2015). Methods for assessment of interlaminar tensile strength of composite materials. J. Compos. Mater..

[52] Cao D., Hu H., Duan Q., Song P., Li S. (2019). Experimental and three-dimensional numerical investigation of matrix cracking and delamination interaction with edge effect of curved composite laminates. Compos. Struct..

[53] Wang Y., Guan Z., Wang X., Liu F., Jiang T., Xu J. (2020). Mechanical properties and damage analysis of C/C-SiC curved beam under four-point bending: Experimental and numerical investigation. Ceram. Int..

